# X-ray Photoelectron Spectroscopy (XPS) Analysis of Ultrafine Au Nanoparticles Supported over Reactively Sputtered TiO_2_ Films

**DOI:** 10.3390/nano12203692

**Published:** 2022-10-21

**Authors:** Zineb Matouk, Mohammad Islam, Monserrat Gutiérrez, Jean-Jacques Pireaux, Amine Achour

**Affiliations:** 1 Technology Innovation Institute, Abu Dhabi P.O. Box 9639, United Arab Emirates; 2 GE Aerospace, 3290 Patterson Ave SE, Grand Rapids, MI 49512, USA; 3 Research Centre, Physics of Matter and Radiation (PMR), LISE Laboratory, University of Namur, B-5000 Namur, Belgium; 4 Pixium Vision S.A. 74 Rue du FGB Saint-Antoine, 75012 Paris, France

**Keywords:** gold nanoparticles, nitrogen-doped titania, molecular oxygen, hydroxyl groups, X-ray photoelectron spectroscopy, anatase TiO_2_

## Abstract

The impact of a titania (TiO_2_) support film surface on the catalytic activity of gold nanoparticles (Au NP) was investigated. Using the reactive dc-magnetron sputtering technique, TiO_2_ films with an amorphous, anatase, and nitrogen-doped anatase crystal structure were produced for a subsequent role as a support material for Au NP. Raman spectra of these TiO_2_ films revealed that both vacuum and NH_3_ annealing treatments promoted amorphous to anatase phase transformation through the presence of a peak in the 513–519 cm^−1^ spectral regime. Furthermore, annealing under NH_3_ flux had an associated blue shift and broadening of the Raman active mode at 1430 cm^−1^, characteristic of an increase in the oxygen vacancies (*V_O_*). For a 3 to 15 s sputter deposition time, the Au NP over TiO_2_ support films were in the 6.7–17.1 nm size range. From X-ray photoelectron spectroscope (XPS) analysis, the absence of any shift in the Au 4f core level peak implied that there was no change in the electronic properties of Au NP. On the other hand, spontaneous hydroxyl (–OH) group adsorption to anatase TiO_2_ support was instantly detected, the magnitude of which was found to be enhanced upon increasing the Au NP loading. Nitrogen-doped anatase TiO_2_ supporting Au NP with ~21.8 nm exhibited a greater extent of molecular oxygen adsorption. The adsorption of both –OH and O_2_ species is believed to take place at the perimeter sites of the Au NP interfacing with the TiO_2_ film. XPS analyses and discussions about the tentative roles of O_2_ and –OH adsorbent species toward Au/TiO_2_ systems corroborate very well with interpretations of density functional theory simulations.

## 1. Introduction

Metal oxide-supported catalyst nanoparticles (MO/C) are one of the most actively pursued catalyst systems for modern chemical applications, including the oxidation of volatile organic compounds, photocatalysis, and energy-harvesting materials [1,2,3,4,5,6]. Gold (Au), platinum (Pt), and ruthenium (Ru) nanoparticles (NP) are among the MO/C system types that possess an unexpectedly high catalytic activity, coupled with optical properties. Besides the type and size of the catalyst nanoparticles, the oxide support, which may be Al_2_O_3_, CeO_2_, Fe_2_O_3,_ or TiO_2_, also plays a significant role [7,8,9,10,11,12]. This was first ascribed to their influence on the stabilization of the active metal catalyst nanoparticles with some better-controlled dispersion and surface area [8,13]. However, it was realized later that some physicochemical interactions between the metal catalysts and their support could greatly influence the catalytic performance of the entire system [8,9,10,11,12,13]. These support/catalyst interactions have the ability to control and tune the catalysts’ crystalline structure and their chemical reactivity.

Although the MO/C has been extensively investigated for high-performance catalytic application, an adequate understanding of the fundamental underlying mechanisms in many applications is still lacking [13,14,15,16]. For instance, TiO_2_-supported Au nanoparticles (Au/TiO_2_) have been studied for decades as a potential catalysis system, especially for CO oxidation at low temperatures. Although the catalytic performance has been improved, a full understanding of the mechanism of CO oxidation at low temperatures (T) is still missing [8,14,15,17,18]. Therefore, any further analyses and modeling of the interactions between all these components are important to develop more performant catalytic systems. For instance, the role of molecular oxygen and water/hydroxyl adsorption were both highlighted to increase the catalytic activity for CO oxidation at low T, particularly in the case of titania and other metal oxide supports [8,15,16,19,20,21]. It was reported that molecular oxygen adsorption leads to lowering the CO to CO_2_ reaction temperature, for example, not only on TiO_2_-supported Au NP but also for other types of catalyst supports and nanoparticles [19,20,21].

Concerning molecular oxygen adsorption (MOA) on titania, Tan et al. [22] demonstrated MOA on a rutile surface using scanning tunneling microscopy. Setvin et al. [23] have also detected electron transfer between anatase TiO_2_ and an O_2_ molecule directly observed by atomic force microscopy. Oxygen adsorption on anatase TiO2 (101) and (001) surfaces from first principles was also studied theoretically by Zeng et al. [24]. When Au NP are deposited on TiO_2_, the MOA is localized at the perimeter of the Au nanoparticles, as demonstrated by Green et al. [8] using DFT and infrared-kinetic measurements. As for water adsorption, it has been studied by Valdés et al. [16], who used density functional theory calculations in the case of TiO_2_ films. These authors found that presence of Au NP favors the hydroxyl accumulation on the anatase surface. To date, direct spectroscopic evidence of the presence of molecular oxygen and hydroxyl groups (on Au NP using, e.g., X-ray photoelectron spectroscopy (XPS), has not yet been demonstrated. The present experimental work validates the theoretical findings made by Valdés et al. [16].

In this work, XPS surface characterization provides direct experimental evidence of the OH and O_2_ (co)adsorption on Au NP supported by pure as well as nitrogen doped TiO_2_. The influence of the Au NP size (sputter deposition time) and composition and structure of the TiO_2_ support films are explored. We conclude on direct experimental evidence of molecular oxygen and hydroxyl adsorptions on TiO_2_-supported Au NP. Such support/NP systems are presently being tested for their effective catalytic properties.

## 2. Experimental

### 2.1. Preparation of Titania Films

Titanium oxide (TiO_2_) films were produced at room temperature over Si (100) wafers, using a direct-current (DC) reactive magnetron sputtering process in a machine equipped with a turbo molecular pump to ensure at least a 10^−7^ mbar base pressure. As sputtering and reactive gases, argon (Ar) and oxygen (O_2_) gases (purity level of ≥99.999%) were used, respectively. The elemental titanium target (99.999%) was sputtered at 250 W power and 5 × 10^−3^ mbar pressure under the Ar and O_2_ flow rates of 23 and 8 sccm, respectively. All the films were deposited for 15 min time. Their film thickness, estimated with a diamond stylus-based contact profilometer, was ∼140 nm for all the samples.

### 2.2. Thermal Treatment

The annealing of the as-deposited TiO_2_ films was performed in the same reactor used for the deposition. The samples were heat treated for 1 h either in a vacuum or under an ammonia (NH_3_) flow at 600 °C. For vacuum annealing, the chamber pressure was maintained at 0.4 mbar, whereas annealing in NH_3_ was carried out at 25 mbar pressure and 10 sccm NH_3_ flux. The as-made TiO_2_, the annealed TiO_2_, and the NH_3_ annealed TiO_2_ are named TiO_2_-amorphous, TiO_2_-Annealed600, and TiO_2_-annealed600-NH_3_, respectively.

### 2.3. Au NP Deposition

**Figure 1:**
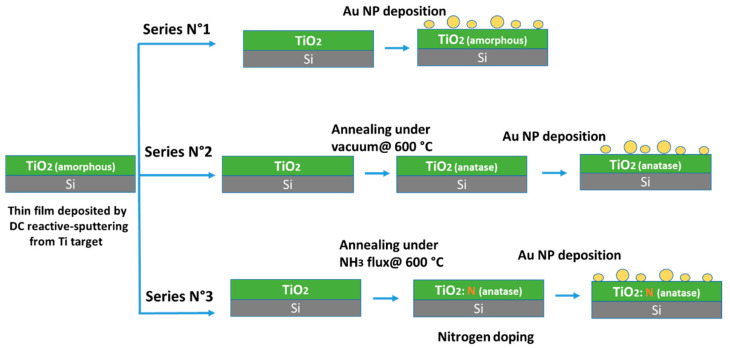
Flow diagram showing all the steps towards synthesis of Au NP-supported TiO_2_ films.

The as-prepared and vacuum-annealed TiO_2_ films were used as supports for Au NP deposition via direct-current (DC) plasma sputtering in a separate chamber. The Au target (≥99.999 purity; 2-in diameter) was sputtered at 9 W power, 5 × 10^−3^ mbar, under 30 sccm Ar flux for 3, 6, 10, or 15 s to obtain different Au loadings or particle sizes. The fabrication steps of the MO/C, including the TiO_2_ film deposition and the heat treatment followed by Au NP deposition on the three TiO_2_ polymorphs, are schematically illustrated in Figure 1.

### 2.4. Materials Characterization

The influence of deposition time on size and distribution of Au NP was assessed by operating a transmission electron microscope (2100F, JEOL, Tokyo, Japan) at 200 keV operating voltage. The samples were prepared by scrapping off some of the Au/TiO_2_ film using diamond tip and preparing a colloidal suspension in acetone for good dispersion. After that, few droplets from the suspension were taken onto perforated carbon-coated copper holey grid.

For phase composition analysis, micro-Raman spectroscopy was performed by means of a Horiba Jobin Yvon LabRam HR system fitted with a liquid-nitrogen-cooled CCD multichannel detector. The spectra were collected under ambient conditions using the 514 nm line of an argon laser. For surface chemical studies, X-ray photoelectron spectroscopy (XPS) measurements were carried out on a K-Alpha instrument (Thermo Scientific, East Grinstead, UK) using a monochromatic X-ray beam (Al Kα) with a spot size of 300 × 300 μm^2^. The spectrometer is equipped with a flood gun for charge compensation, any charge-induced energy shift being corrected by fixing the C 1s line at 284.4 eV. For the peak-fitting procedure, a Shirley-type background was subtracted from the spectra while the peaks were fitted with symmetric Gaussian functions.

## 3. Results and Discussion

### 3.1. Characterization of the TiO_2_ Films and Au NP

**Figure 2:**
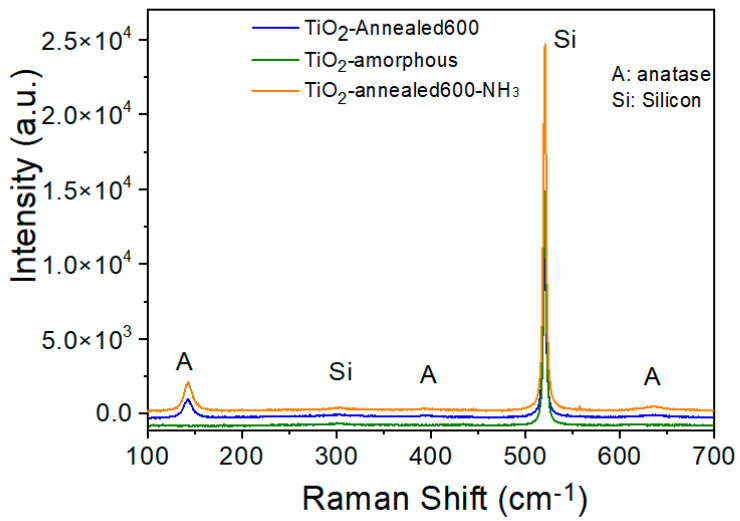
Raman spectra of the TiO_2_ films with amorphous, annealed, and N-doped annealed phases.

The Raman spectra of the three different TiO_2_ films are shown in Figure 2. Due to their small thickness, all the samples exhibit a strong peak at ~520 cm^−1^ and another weak peak at ~303 cm^−1^ that have to be associated with the Si substrate [25]. The as-deposited TiO_2_ film was found to have an amorphous structure, as indicated by the absence of any peak’s characteristic of the TiO_2_ phase. Upon annealing at 600 °C, either in vacuum or under NH_3_ flow, TiO_2_ film crystallization occurred from the amorphous to the anatase phase; indeed, the Raman spectra evidenced peaks positioned at 142.3, 394.4, and 634.9 cm^−1^ that may be assigned to the E_g_ and B_1g_ vibration modes of the anatase structure [26,27]. Although the A_1g_/B_1g_ vibration mode located in the 513–519 cm^−1^ range cannot be distinguished because of an overlap with the Si substrate peak, there is indeed in this wavelength a region peak broadening to varying extents, indicating the anatase phase formation. It is known that thermal annealing at temperatures in the range of 450 to 800 °C does induce an amorphous to anatase phase transformation [28]. The Raman spectra of the different TiO_2_ films did not show any peak associated with either the rutile or the brookite TiO_2_ polymorphs [27,29,30].

In the case of the TiO_2_ sample annealed under NH_3_ flux, a small blue shift (toward the higher wavenumber region) and a little increase in the full-width at half maximum (FWHM) were observed at 143.0 cm^-1^. These broadening and shifts of Raman active modes can be assigned to the presence of oxygen vacancies (*Vo* lattice defects) generated by the displacement of oxide ions from the TiO_2_ crystal lattice [31]. Both shift and peak broadening demonstrate that oxygen vacancies (*V_O_*) are more numerous in the case of the TiO_2_-annealed600-NH_3_ sample. The present Raman analysis suggests an increase in the oxygen vacancies through nitrogen doping.

**Figure 3:**
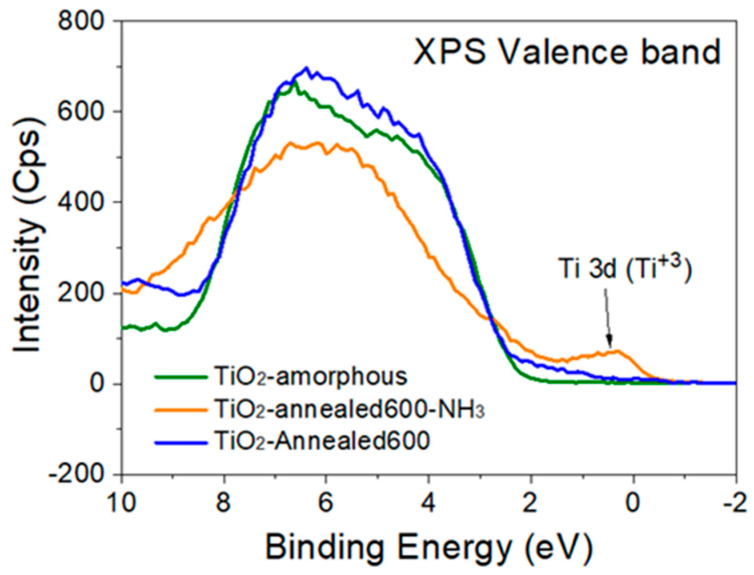
X-ray photoelectron spectroscope valence band spectra of different titania films.

The XPS valence band spectra of the three TiO_2_ films are presented in Figure 3. The TiO_2_-annealed600-NH_3_ sample shows a band gap spectrum markedly different from the other samples with the presence of a clear and intense band close to the Fermi-level. This band culminating at ~0.84 eV can be attributed either to the Ti 3d (Ti^3+^) defect states related to the *V_O_*, or to Ti interstitial defects in the TiO_2_ lattice [32]. This peak also confirms that annealing under NH_3_ flux leads to the generation of surface defects such as oxygen vacancies, in good agreement with the results of the Raman analysis of the same films.

Upon curve fitting of the N 1s XPS core level spectrum, appears a β-N peak representative of the nitrogen doping via substitution at or near the surface (Figure S1). The generation of oxygen vacancies in the TiO_2_ anatase phase upon doping with nitrogen was also reported earlier [33,34,35]. The surface of the TiO_2_-annealed600-NH_3_ film should, therefore, contain a considerable amount of oxygen vacancies. Nitrogen incorporation into the TiO_2_ lattice was also quantitatively confirmed using the XPS survey spectrum of the TiO_2_-annealed600-NH_3_ film (Figure S2), showing a nitrogen content of 18.5 at.% besides the other detected elements; namely, carbon, titanium, and oxygen. The atomic percent values of Ti, O, C and N in different TiO_2_ films, calculated from XPS spectra, have been given in Table S1.

**Figure 4:**
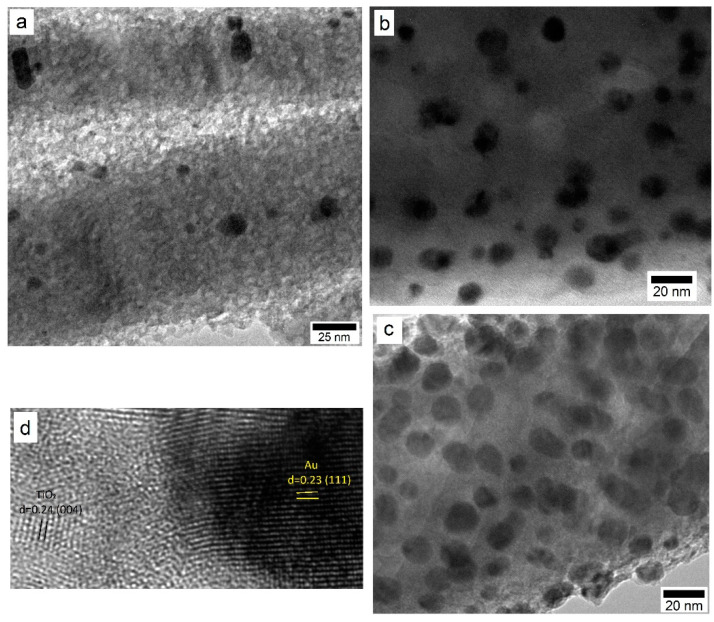
TEM micrographs of Au/TiO_2_ system with Au NP from sputter deposition for (**a**) 3 s, (**b**) 6 s, and (**c**) 10 s. (**d**) Lattice structure of Au NP and TiO_2_ film.

The Au NP size and distribution over TiO_2_ for 3, 6, and 15 s deposition times were investigated using TEM. The information about the average size and distribution was extracted from high magnification micrographs shown in Figure 4. While a relatively low deposition time of 3 s indicated the presence of Au NP that were far apart from each other, there was some degree of cluster formation among them. The average Au NP size was estimated to be ~6.7 ± 1.4 nm. As the deposition time was increased to 6 s, the average size increased to 13.8 ± 1.8 nm, with an associated decrease in the inter-particle spacing. For the 10 s deposition, the average Au NP size was estimated to be ~17.1 ± 2.0 nm. At a high magnification (Figure 4d), the lattice structures were indexed to be TiO_2_ and Au with respective d and (hkl) values of 0.24 nm for (004) and 0.23 nm for (111).

### 3.2. Effect of Au NP Loading

The XPS Au 4f core level spectra of the Au catalyst nanoparticles deposited over different TiO_2_ polymorph films, with different loading levels/sizes, are showcased in Figure S3. In all the samples, the Au 4f peak 7/2 positioned at 84.1–84.0 eV is associated with gold in its metallic state [36]. The fact that there was no shift (no more than 0.1 eV) in the Au 4f peak position, suggests there is no significant chemical interaction between the deposited Au particles and their TiO_2_ support. In other words, the crystal structure of the TiO_2_ film does not influence the interaction between the Au NP and the support. However, the degree of Au nanoparticles loading onto TiO_2_ does affect the extent of oxygen and hydroxyl group co-adsorption, as will be shown in the next sections.

#### 3.2.1. Amorphous TiO_2_ Support

**Figure 5:**
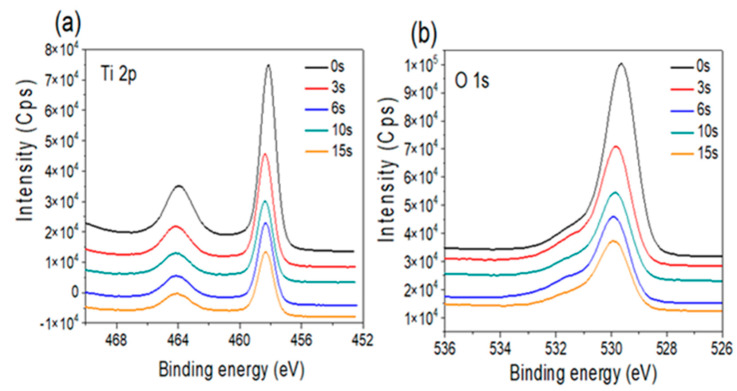
X-ray photoelectron spectroscope data for the amorphous titania film after Au NP deposition for 0 to 15 min: (**a**) Ti 2p core level spectra, and (**b**) O 1s core level spectra.

The high-resolution XPS spectra of the Ti2p and O1s core levels for the TiO_2_-amorphous sample, loaded with Au NP (3 to 15 s deposition time), are presented in Figure 5a,b, respectively. All the samples are characterized by similar Ti 2p XPS spectra, with the Ti^4+^ (1/2, 3/2) spin–orbit doublet components at ~458.1 and 463.9 eV binding energies, showing a small shift toward high binding energies for higher Au NP loading levels [3]. The O 1s core level XPS spectra (Figure 5b) are positioned around 529.2 eV, a peak that is usually assigned to Ti^4+^–O bonds in the TiO_2_ lattice. As the peak intensity, expected to be proportional to the number of oxygen atoms surrounding the oxidized titanium [3], appears very constant for this TiO_2_-amorphous surface, one concludes that a change in the Au NP loading does not have any influence on the stoichiometry of the support.

#### 3.2.2. Annealed Titania Film

**Figure 6:**
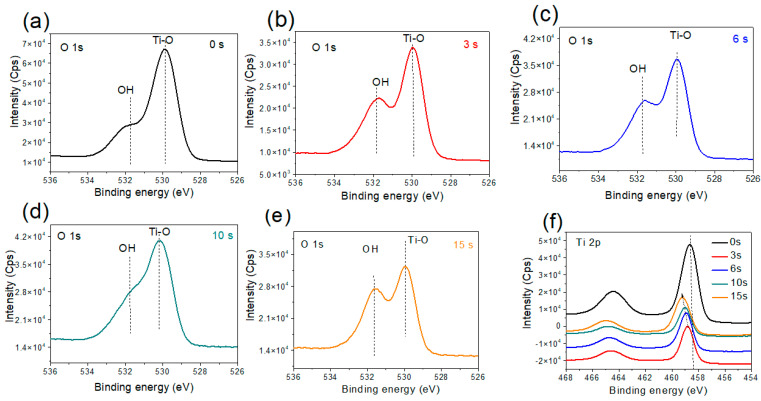
X-ray photoelectron spectroscope data for the 600 °C annealed titania film (TiO_2_-annealed600) after Au NP deposition for 0 to 15 s: (**a**–**e**) O 1s core level spectra, and (**f**) Ti 2p core level spectra.

The high-resolution O1s spectra of the TiO_2_-Anneal600 sample without and with different Au NP loadings are presented in Figure 6. For all the samples, the XPS envelope is constituted of two distinct component peaks; a strong one at 529.9 eV that may be assigned to the Ti^4+^–O chemical bond, and a shoulder peak (with variable intensities depending on the amount of Au NP) located at 531.8 eV that can be attributed to the presence of hydroxyl groups (OH) [3,37]. Interestingly, the intensity of the OH peak increases with the increase in the Au NP loading, with no apparent shift in the binding energy of Au NP (Figure S3 and S4). In the high-resolution O1s XPS fitting spectra before and after 15 s of Au NP deposition time (Figure S5), an increase in the OH component is by ~25 %. This suggests that increasing the Au NP loading on the TiO_2_-Anneal600 surface with the anatase phase structure induces hydroxyl groups attachment on the surface, most likely at the perimeter of the Au particles since no surface oxidation of the Au NP is observed. This conclusion is also supported by a recent report using density functional theory (DFT) to demonstrate that the presence of Au NP on the anatase TiO_2_(101) surface favors hydroxyl groups accumulation precisely at the Au/TiO_2_ periphery [16]. Therefore, it appears that our XPS analysis is the first, direct experimental evidence of this hypothesis; one should add that Valdé et al. [16] also calculated that the OH groups adsorption is associated with an easy electron transfer from the deposited gold clusters to the OH groups at the TiO_2_ surface. Another possible hypothesis is the titania support can act as a template for the crystallization of gold. Therefore, the Au NP adopt the crystal structure of the support, and thus the marked difference between the hydroxyl group adsorption in the case of Au NP on the surface of TiO_2_ anatase and amorphous phases could also be due to inducing structure in the Au NP by the surface. In other words, for the two cases studied, as the Au NP surfaces have different crystal structures, they have different affinities for the adsorption of hydroxyl groups, such as those reported in the case of the mechanisms of nucleation and solid–solid-phase transitions in triblock Janus assemblies [38,39].

The high-resolution XPS spectra of the Ti2p core level for the TiO_2_-Anneal600 samples with different Au NP loadings are shown in Figure 6f. In contrast to the TiO_2_-amorphous sample (Figure 5a), one observes a progressive shift of the Ti2p line to high binding energies when the Au NP deposition time increases; this suggests a higher positive oxidation state, i.e., Ti^4+^ owing to extra coulombic interaction between the ionic cores and the photo-emitted electrons along with the possibility of hydroxyl species attachment on the TiO_2_-Anneal600 sample surface. Upon binding to the TiO_2_ surface, the OH groups strongly attract electrons, which agrees with the high-resolution XPS O1s and Ti2p analyses.

**Figure 7:**
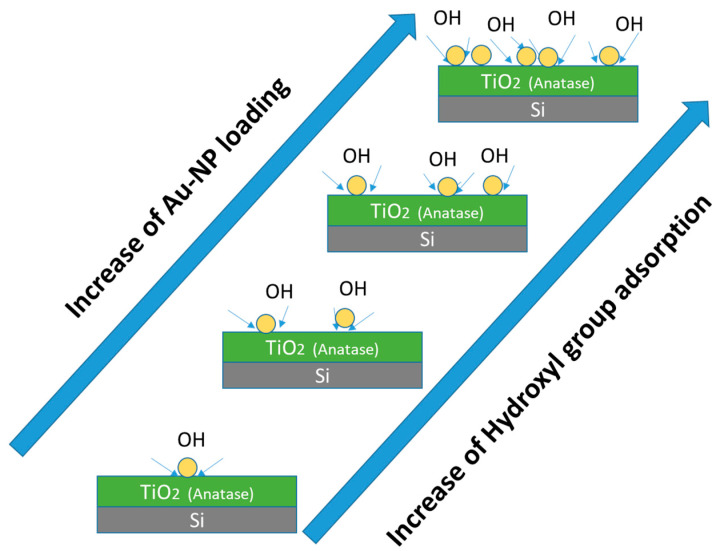
Schematic illustration of the effect of Au NP loading on the extent of hydroxyl group attachment to the anatase TiO_2_ film surface.

This particularity of the Au/TiO_2_-Annealeded600 sample does not only confirm the theoretical prediction of Au NP favoring OH groups attachment on the anatase surface, but also suggests an increase in the extent of OH attachment with greater Au NP loading, as suggested by the schematic diagram in Figure 7. One should add that such a phenomenon happens exclusively in the case of the TiO_2_ anatase phase but is not observed in the case of the TiO_2_-amorphous sample. Further experimentation is required to explore the possibility of similar behavior in rutile or brookite TiO_2_ polymorphic surfaces, which is beyond the scope of the present work.

According to our understanding developed from XPS analysis, the OH content on the anatase TiO_2_ surface increases with an increase in the Au NPs on the surface (loading level effect). It is speculated that the OH attachment takes place at the perimeter of the Au NPs onto the TiO_2_-Anneal600 interface. These results are expected to generate great implications towards the development of more performant Au/TiO_2_ catalyst systems operating at a low temperature [40,41].

#### 3.2.3. Annealed, Doped Anatase TiO_2_ Support

**Figure 8:**
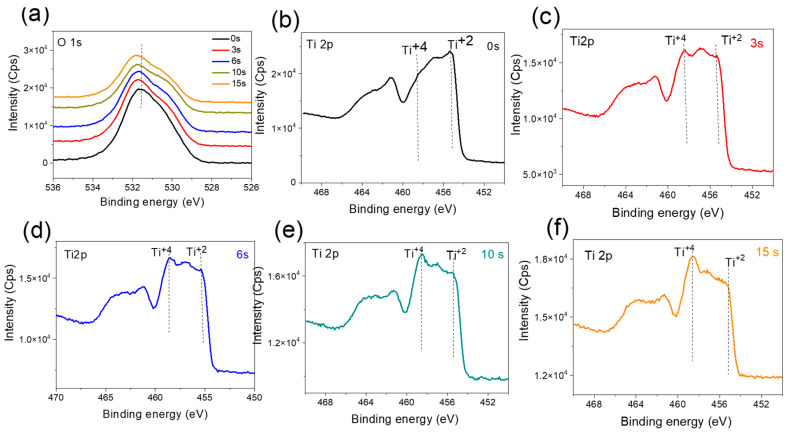
XPS data for nitrogen-doped, annealed TiO_2_ film (TiO_2_-annealed600-NH_3_) before and after NP deposition for 3–15 s): (**a**) O 1s core level spectra and (**b**–**f**) Ti 2p core level spectra.

The high-resolution O1s core level XPS spectra of the TiO_2_-annealed600-NH_3_ samples supporting different loadings of Au NPs are presented in Figure 8a. Immediately, the shape of the O1s envelope indicates an abundant presence of oxygen vacancies and OH groups, while the varying Au NPs did not seem to influence the different components of the O 1s peaks (fitted spectra are presented in Figure S7).

The high-resolution XPS spectra of the Ti2p core level for the TiO_2_-annealed600-NH_3_ samples with different Au NPs loadings are presented in Figure 8b–f. As compared to the TiO_2_-amorphous and TiO_2_-Anneal600 samples, the shape of the Ti2p core level for the doped anatase sample is completely different. The presence of the Ti^2+^ and Ti^3+^, along with Ti^4+^, indicates the film surface to be highly doped with nitrogen. Indeed, the shape of the Ti2p envelop resembles that of TiON [42], although the bulk is of the anatase phase composition, as confirmed from the Raman spectroscopy. Quite interestingly, the Au NPs deposition over the TiO_2_-annealed600-NH_3_ film leads to the increase in the Ti^3+^ and Ti^4+^ components upon increasing the Au NPs deposition time, suggesting oxidation of the resulting surface [42]. Since the analysis of the Au 4f high-resolution spectra did not indicate any Au NPs oxidation (Figure S3), this implies that oxidation takes place at the perimeter site of the Au NPs on the TiO_2_-annealed600-NH_3_ interface. This finding is also supported by the fact that the comparison of the XPS Ti2p spectra of the TiO_2_-annealed600-NH_3_ before and after 15 s Au NPs deposition (Figure S6) demonstrates the Ti^4+^ increase for a relatively high Au deposition time. The oxidation of the TiO_2_-annealed600-NH_3_ is believed to take place at the interfacial region with the Au NPs, because of the molecular oxygen adsorption (MOA). MOA has already been reported in the case of TiO_2_-supported Au NPs [8,16,20,43], and is ascribed to be activated by the Au NPs at the perimeter of the Au/TiO_2_ interface. Besides that, the presence of the supported Au NPs strongly stabilizes the adsorption of O_2_. In the present study, the XPS analysis reveals a reduction of the anatase phase due to heavy nitrogen doping at the film surface region. Other investigations related to MOA on the Au NPs supported by reduced TiO_2_ are scarce, due to the high complexity of the DFT calculations. Nonetheless, Yon et al. [44] found that the Au clusters remain either neutral or acquire a positive charge; therefore, the intuitively expected electron transfer from the oxygen vacancy to the gold cluster can be safely ruled out. In our case, the adsorbed O_2_ does not seem to fill the oxygen vacancies since the curve fitting of the XPS O1s spectra of the TiO_2_-annealed600-NH_3_ samples (Figure S7), before and after Au NPs deposition, does not exhibit any significant decrease in the component peak related to the oxygen vacancies.

**Figure 9:**
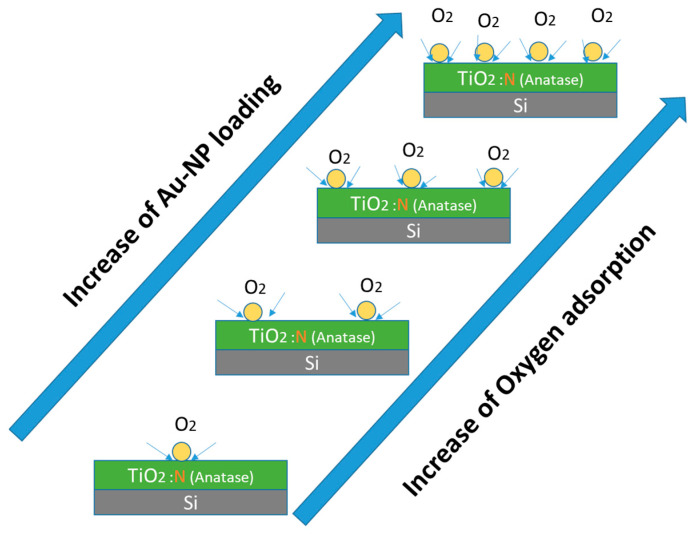
Schematic representation of the effect of Au NP loading on the extent of molecular oxygen attachment to the nitrogen-doped anatase TiO_2_ film surface.

Furthermore, the high-resolution XPS N1s spectra (Figure S8) concur to indicate there is no decrease in the β-N component peak intensity upon Au NPs deposition; this clearly implies the presence of oxygen vacancies in the N-doped TiO_2_ even after Au NP deposition. In that case, therefore, against the general intuition, the O_2_ adsorption (MOA) must primarily happen to be at the Au/TiO_2_-annealed600-NH_3_ perimeter, as the adsorbed oxygen species do not occupy oxygen vacancies present on the TiO_2_ film surface. Figure 9 is a schematic illustration of the effect of Au NPs loading on the MOA extent.

## 4. Conclusions

While reactive sputtering of TiO_2_ leads to the formation of an amorphous structure in the as-deposited film, vacuum annealing under controlled conditions causes transformation to an anatase crystal structure. The anatase phase formation is also confirmed from Raman spectra via the presence of E_g_ and B_1g_ active vibration modes positioned at 142.3, 394.4, and 634.9 cm^−1^. By carrying out sputter deposition for 3 to 15 s, Au NP with an average size in the range of 7.7 to 17.1 nm can be produced over TiO_2_ support films. The crystal structure of the TiO_2_ support film does not seem to influence chemical bonding of Au NP in the sense that no shift in the Au 4f peak position was observed during XPS analysis.

From extensive XPS investigations, it was found that any change in the Au NP loading over amorphous TiO_2_ did not affect adsorption behavior and surface chemistry of the resulting MO/C system. On the contrary, increasing the Au NP size over anatase leads to a greater extent of hydroxyl group (OH) attachment, which agrees with density function theory predictions. While annealing at 600 °C under NH_3_ flow produced a nitrogen-doped anatase phase TiO_2_ with high density of oxygen vacancies at and near surface regions, an increase in the Au NP loading enhanced the degree of molecular oxygen adsorption presumably at the perimeter interface of Au/TiO_2_-anatase. The observation that the surface density of oxygen vacancies did not decrease upon MOA, if counterintuitive, implies that the O_2_ species attach at the Au/TiO_2_ interfacial region and not at the oxygen vacancies sites.

In a nutshell, this work can be considered as direct experimental evidence for OH and O_2_ adsorption on Au NP supported by the anatase phase or anatase with oxygen vacancies. The control of the OH and O_2_ contents at the Au/TiO_2_ surface is expected to become an efficient parameter to be optimized to improve the performance of this Au/TiO_2_ system as a photocatalyst and a potential catalyst for VOC degradation at low temperature.

## Data Availability

Not applicable.

## Supplementary Materials

The following supporting information can be downloaded at: https://www.mdpi.com/article/10.3390/nano12203692/s1, Table S1: Elemental composition of the TiO_2_ -amorphous, TiO_2_ -Annealed600 and TiO_2_-annealed600-NH_3_ films from XPS analysis; Figure S1: XPS N1s fitted core level spectrum of the TiO_2_-annealed600-NH_3_ film; Figure S2: XPS survey spectra of the TiO_2_ -amorphous, TiO_2_ -Annealed600 and TiO_2_-annealed600-NH_3_ films; Figure S3: Au 4f core level spectra of the Au NPs deposited onto the (a) TiO_2_ -amorphous, (b) TiO_2_ -Annealed600 (c) TiO2-annealed600-NH3 films; Figure S4: XPS valence band spectra of the samples obtained for the Au NPs deposited onto the TiO2 -Annealed600 film during different deposition times (3 to 15 s). Similar results are obtained in the case of the Au NPs deposited onto the TiO_2_ -amorphous or TiO_2_-annealed600-NH_3_ films; Figure S5: XPS O1s fitted core level spectra of the TiO_2_ -Annealed600 film before (a) and after (b) 15 s Au NPs deposition; Figure S6: XPS Ti2p fitted core level spectra of the TiO_2_ -Annealed600-NH_3_ film before (a) and after (b) 10 s Au NPs deposition; Figure S7: XPS O1s fitted core level spectra of the TiO_2_ -Annealed600-NH_3_ filmbefore (a) and after (b) 15 s Au NPs deposition; Figure S8: XPS N1s fitted core level spectra of the TiO_2_ -Annealed600-NH_3_ film before (a) and after (b) 15 s Au NPs deposition. References [45,46] are cited in the Supplementary Materials.
