# Supplementary material for: X-ray Photoelectron Spectroscopy (XPS) Analysis of Ultrafine Au Nanoparticles Supported over Reactively Sputtered TiO2 Films
Source: Nanomaterials (Basel). 2022 Oct 21;12(20):3692. doi: 10.3390/nano12203692 (PMC9609015; doi:10.3390/nano12203692)
Supplement: Supplementary file 1 [file nanomaterials-12-03692-s001.zip › nanomaterials-1957906-supplementary.pdf]

Supplementary Information

X-ray Photoelectron Spectroscopy (XPS) Analysis of Ultrafine Au Nanoparticles Supported over Reactively Sputtered TiO_2 Films

Zineb Matouk [1], Mohammad Islam [2,*], Monserrat Gutiérrez [1], Jean-Jacques Pireaux [3], Amine Achour [4,*]

[1] Technology Innovation Institute, Abu Dhabi P.O. Box 9639, United Arab Emirates; zineb.matouk@tii.ae (Z.M.); monserrat.gutierrez@tii.ae (M.G.)

[2] GE Aerospace, 3290 Patterson Ave SE, Grand Rapids, MI 49512, USA

[3] Research Centre, Physics of Matter and Radiation (PMR), LISE Laboratory, University of Namur,
B-5000 Namur, Belgium; jean-jacques.pireaux@unamur.be

[4] Pixium Vision S.A. 74 Rue du FGB Saint-Antoine, 75012 Paris, France

* Correspondence: mohammad.islam@gmail.com (M.I.); aachour@pixium-vision.com (A.A.)

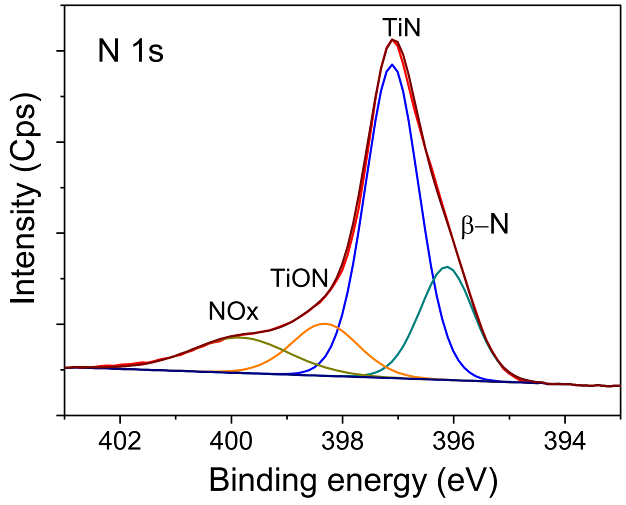

Figure S1. XPS N1s fitted core level spectrum of the TiO_2-annealed600-NH_3 film.

The fitted XPS N1s core level spectrum of the TiO_2-annealed600-NH_3 film shows the presence of B-N at the low binding energy. This confirms the surface of this film is doped by nitrogen and therefore, one can deduce the presence of oxygen vacancies [1–2]

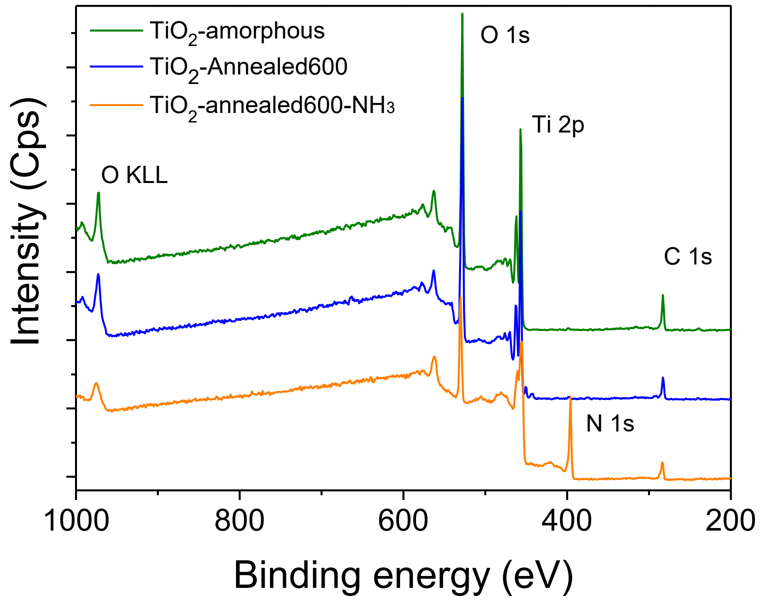

Figure S2. XPS survey spectra of the TiO_2-amorphous, TiO_2-Annealed600 and TiO_2-annealed600-NH_3 films

The XPS survey spectra of all prepared films are presented in Figure S2. The spectra show the presence of titanium, oxygen and carbon elements (adsorbed carbon). In addition, the XPS spectrum of theTiO_2-annealed600-NH_3 film shows an additional intense peak related to nitrogen (the amount is 18.5 at.%). This indicates that the surface of this film is highly doped with nitrogen.

The elemental compositions (from XPS analysis) of the TiO_2-amorphous, TiO_2-Annealed600 and TiO_2-annealed600-NH_3 films are summarized in Table S1.

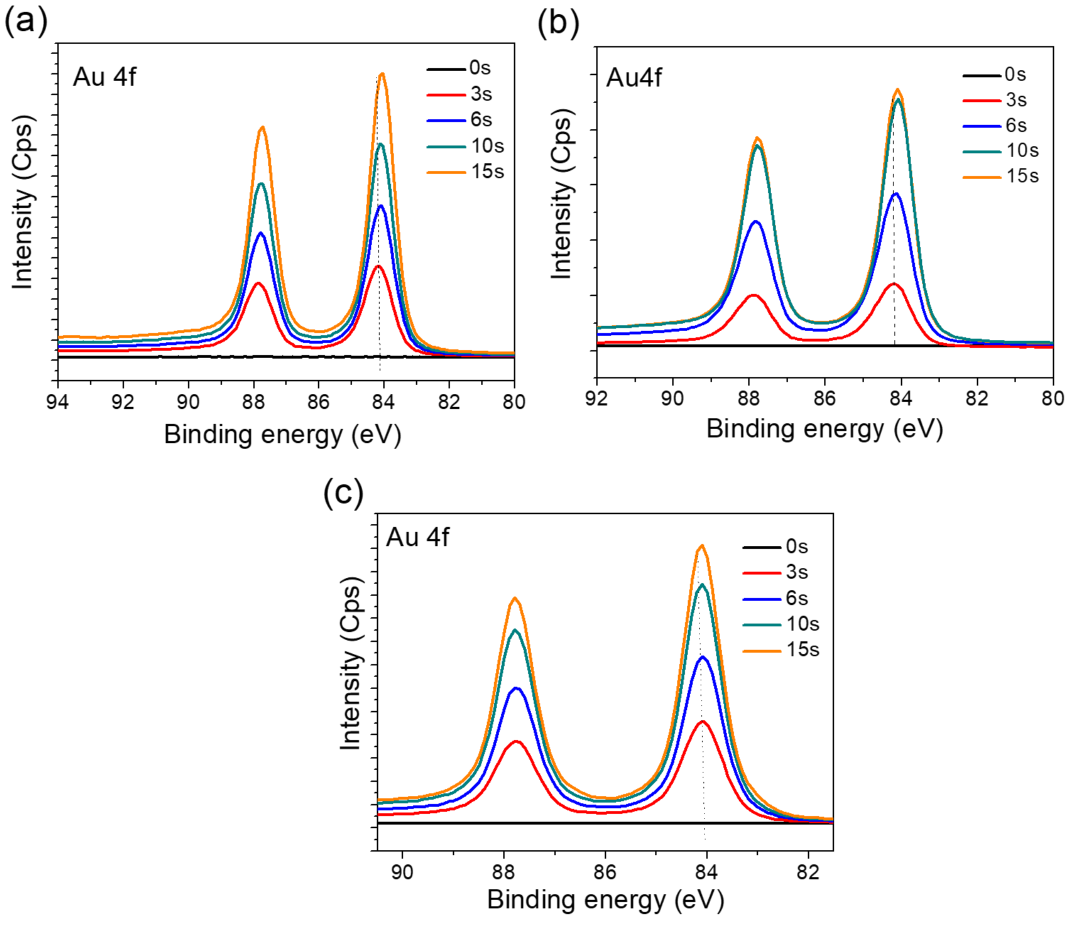

Figure S3. Au 4f core level spectra of the Au NPs deposited onto the (**a**) TiO_2-amorphous, (**b**) TiO_2-Annealed600 (**c**) TiO_2-annealed600-NH_3 films

The Au 4f core level spectra of the Au NPs deposited onto the three TiO_2 polymorph films, show a shift of around 0.1 eV. Such a shift may be considered as negligible to suggest an oxidation, but could eventually be attributed to size effect of the particles.

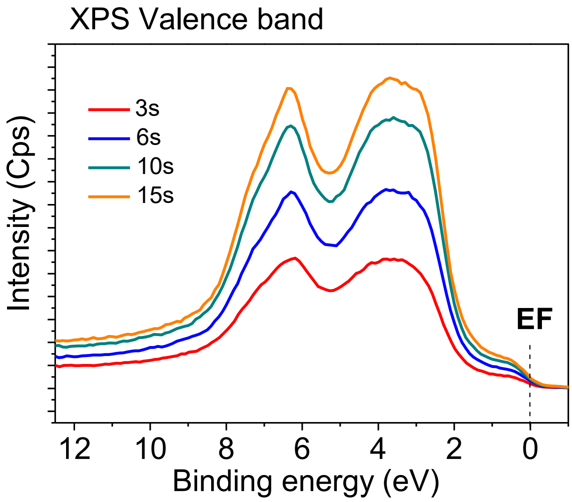

Figure S4. XPS valence band spectra of the samples obtained for the Au NPs deposited onto the TiO_2-Annealed600 film during different deposition times (3 to 15s). Similar results are obtained in the case of the Au NPs deposited onto the TiO_2-amorphous or TiO_2-annealed600-NH_3 films.

The XPS valence band spectra of the Au NPs on the TiO_2¬-Annealed600 film during different deposition times (3-15s) show that the fermi level does not shift whatever the Au deposition time; this confirms no surface oxidation and the chemical stability of the deposited Au NPs

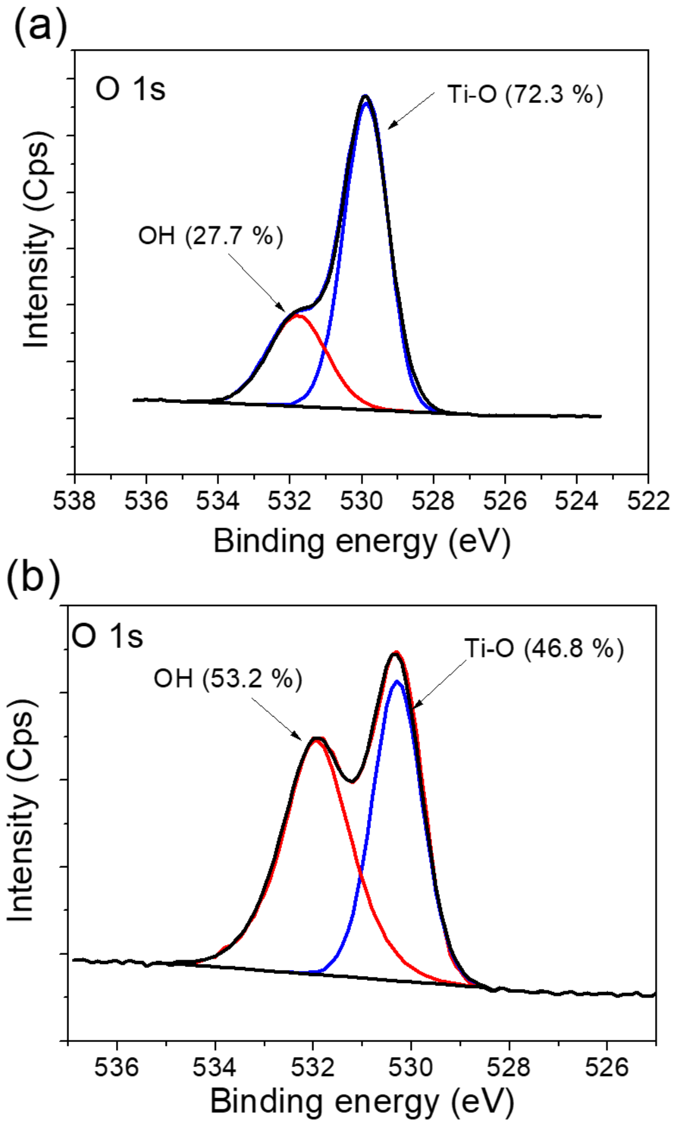

Figure S5. XPS O1s fitted core level spectra of the TiO_2-Annealed600 film before (**a**) and after (**b**) 15 s Au NPs deposition

The XPS O1s fitted core level spectra of the TiO_2¬-Annealed600 film before and after 15 s Au NPs deposition (Fig S5 a and b, respectively) show an increase of the amount of hydroxyl group by almost two-fold when Au NPs are deposited during 15 s.

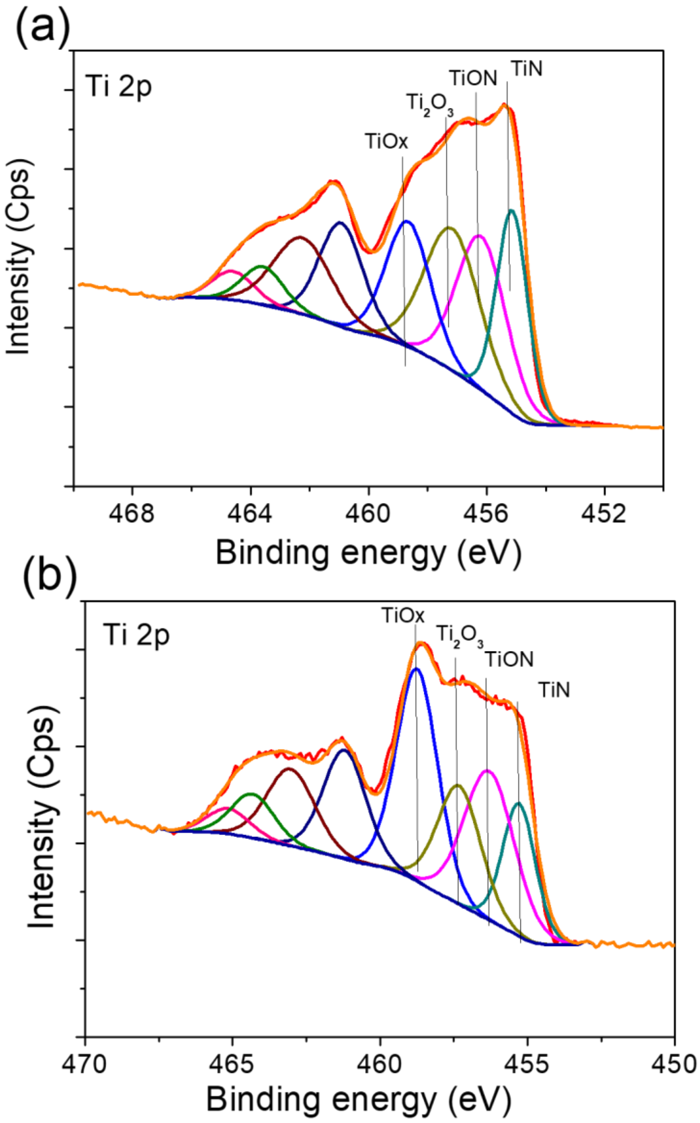

Figure S6. XPS Ti2p fitted core level spectra of the TiO_2-Annealed600-NH_3 film before (**a**) and after (**b**) 10 s Au NPs deposition

The XPS Ti2p fitted core level spectra of the TiO2-Annealed600-NH3 film before and after 15 s Au NPs deposition (Fig S6 a and b, respectively) show clearly an increase of the oxide component (TiOx) and the decrease of the TiN component after Au NP deposition after 15 s. This confirms surface oxidation of the film after Au NP deposition.

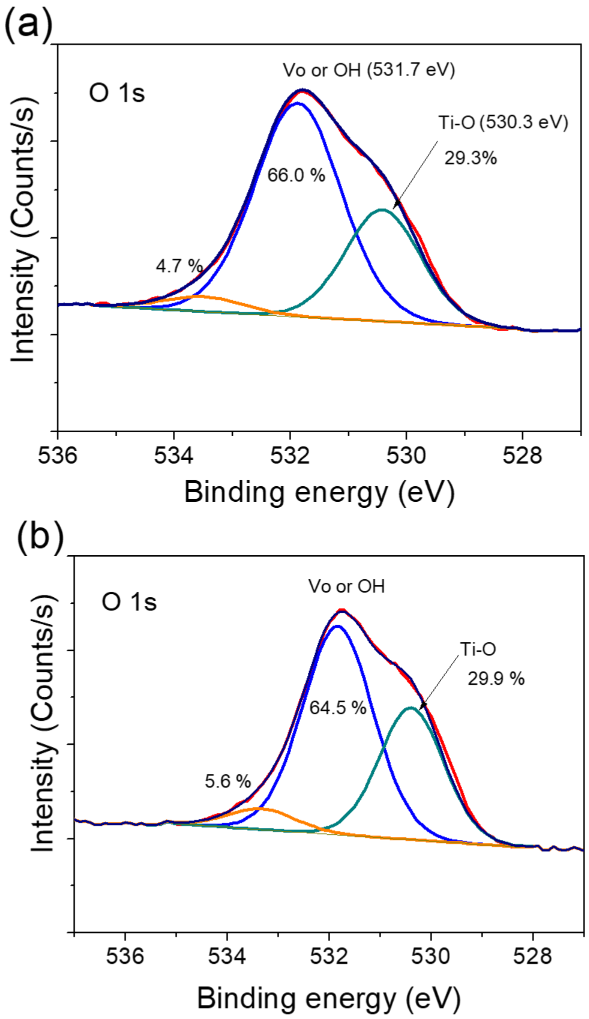

Figure S7. XPS O1s fitted core level spectra of the TiO_2-Annealed600-NH_3 filmbefore (**a**) and after (**b**) 15 s Au NPs deposition

The XPS O1s fitted core level spectra of the TiO_{2}¬-Annealed600-NH3 film before and after 15 s Au NPs deposition show a very small variation of the components that can be related to oxygen vacancies (Vo) or OH groups. This indicates that the oxidation of the film does not take place at the Vo sites.

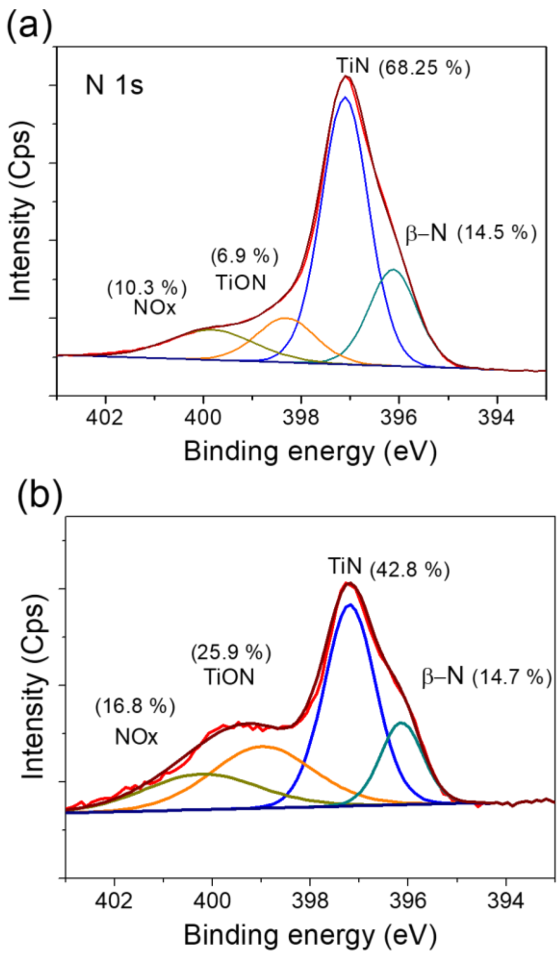

Figure S8. XPS N1s fitted core level spectra of the TiO_2-Annealed600-NH_3 film before (**a**) and after (**b**) 15 s Au NPs deposition

In Figure S8 are shown the XPS N1s fitted core level spectra of the TiO_2-Annealed600-NH_3 film before and after 15 s Au NPs deposition. The atomic percentage of the amount of B-N is almost the same before and after Au NPs deposition, indicating again that no oxidation takes place at the Vo. This is in good agreement with the O1s spectra presented in Figure S7.

Table S1. Elemental composition of the TiO_2-amorphous, TiO_2-Annealed600 and TiO_2-annealed600-NH_3 films from XPS analysis.

Sample	O 1s (at. %)	Ti 2p (at. %)	C 1s (at. %)	N 1s (at. %)
TiO_2-amorphous	41.9	50.7	7.3	×
TiO_2-Anneal600	47.8	46.7	5.3	x
TiO_2-annealed600-NH_3	22.68	53.8	4.9	18.5

References

1. Achour, A.; Porto, R.L.; Soussou, M.-A.; Islam, M.; Boujtita, M.; Aissa, K.A.; le Brizoual, L.; Djouadi, A.; Brousse, T. Titanium nitride films for micro-supercapacitors: Effect of surface chemistry and film morphology on the capacitance. *J. Power Sources* **2015**, *300*, 525–532.
2. Achour, A.; Chaker, M.; Achour, H.; Arman, A.; Islam, M.; Mardani, M.; Boujtita, M.; le Brizoual, L.; Djouadi, M.A.; Brousse, T. Role of nitrogen doping at the surface of titanium nitride thin films towards capacitive charge storage enhancement. *J. Power Sources* **2017**, *359*, 349–354.
